# Transcatheter aortic valve implantation and surgical aortic valve replacement among hospitalized patients with and without type 2 diabetes mellitus in Spain (2014–2015)

**DOI:** 10.1186/s12933-017-0631-6

**Published:** 2017-11-09

**Authors:** Manuel Mendez-Bailon, Noel Lorenzo-Villalba, Nuria Muñoz-Rivas, Jose Maria de Miguel-Yanes, Javier De Miguel-Diez, Josep Comín-Colet, Valentin Hernandez-Barrera, Rodrigo Jimenez-Garcia, Ana Lopez-de-Andres

**Affiliations:** 1 Internal Medicine Department, Instituto de Investigación Cardiovascular, Hospital Clínico San Carlos, Complutense University, Madrid, Spain; 2 Service de Médicine Interne et Cancerlogie, Centre Hospitalier Saint Cyr, Lyon, France; 3 Internal Medicine Department, Hospital Universitario Infanta Leonor, Madrid, Spain; 4 Internal Medicine Department, Hospital General Universitario Gregorio Marañon, Madrid, Spain; 5 Pneumology Department, Hospital General Universitario Gregorio Marañon, Madrid, Spain; 6 Department of Cardiology, Hospital Universitario de Bellvitge, Barcelona, Spain; 7 Preventive Medicine and Public Health Teaching and Research Unit, Health Sciences Faculty, Rey Juan Carlos University, Avda. de Atenas s/n, 28922 Alcorcón, Madrid, Spain

**Keywords:** Type 2 diabetes, Transcatheter aortic valve implantation, Surgical aortic valve replacement, Hospital, Mortality

## Abstract

**Background:**

Type 2 diabetes mellitus (T2DM) is strongly related to the in-hospital and short-term prognosis in patients with cardiovascular diseases needing surgical or invasive interventions. How T2DM might influence the treatment of aortic stenosis (AS) has not been completely elucidated for surgical aortic valve replacement (SAVR) or transcatheter aortic valve implantation (TAVI). The aims of this study were: (1) to describe the use of aortic valve replacement procedures (TAVI and SAVR) among hospitalized patients with and without T2DM; and (2) to identify factors associated with in hospital mortality (IHM) among patients undergoing these procedures.

**Methods:**

We analyzed data from the Spanish National Hospital Discharge Database between January 1, 2014 and December 31, 2015 for patients aged ≥ 40 years. We selected patients whose medical procedures included TAVI (ICD-9-CM codes 35.05, 35.06) and SAVR (ICD-9-CM codes 35.21, 35.22). We stratified each cohort by diabetes status: T2DM (ICD-9-CM codes 250.x0, 250.x2) and no diabetes. We retrieved data about specific comorbidities, risk factors, procedures, and specific in-hospital postoperative complications. Hospital outcome variables included IHM, and length of hospital stay (LOHS).

**Results:**

We identified a total of 2141 and 16,013 patients who underwent TAVI (n = 715; 33.39% with T2DM) and SAVR (n = 4057; 25.33% with T2DM). In patients who underwent TAVI we found no differences in IHM (3.64% in T2DM vs. 5.12% in non-T2DM, p = 0.603). In the cohort of SAVR, mean LOHS was significantly lower in patients with T2DM than in non-diabetic patients (13.77 vs. 17.27 days). IHM was lower in patients with T2DM (4.36% vs. 6.31%, p < 0.01). After multivariable adjustment for both procedures, patients with T2DM had significantly lower IHM than patients without diabetes (adjusted OR 0.60; IC 95% 0.37–0.99 for TAVI and adjusted OR 0.80; IC 95% 0.66-0-96 for SAVR).

**Conclusions:**

T2DM diabetic patients with AS undergoing a valvular replacement procedure through SAVR or TAVI did not have a worse prognosis compared to non-diabetic patients during hospitalization, showing lower IHM after multivariable adjustment. However, given the limitations of administrative data more prospective studies and clinical trials aimed at evaluating the influence of these procedures in diabetic patients with AS are needed.

**Electronic supplementary material:**

The online version of this article (10.1186/s12933-017-0631-6) contains supplementary material, which is available to authorized users.

## Background

Type 2 diabetes mellitus (T2DM) is associated with a wide range of complications and higher risk of cardiovascular death worldwide [1]. Valvular heart disease, in particular is more common in diabetes [2].

Aortic stenosis (AS) is a degenerative valvular disease more related to advance age and the presence of cardiovascular risk factors [3]. In recent studies, such as the CANHEART aortic stenosis study, T2DM was found to be second only to hypertension as the medical condition most associated with aortic stenosis in a population of 1.12 million individuals followed prospectively over 13 years [4].

The association between T2DM and AS is due to the increased development of atherosclerosis in diabetic patients [5]. The activation of the rennin-angiotensin-aldosterone axis, elevation of inflammatory interleukins, production of free radicals, and glycosylation of proteins lead to an increase in profibrotic and calcific processes causing aortic valvular calcification and progression to AS [5, 6].

With the aging of the population, the incidence and prevalence of AS are expected to increase [7]. AS is presently the most common valvular condition leading to valvular replacement [6]. AS has been classically managed with surgical aortic valve replacement (SAVR), but this has changed recently as less invasive procedures such as transcatheter aortic valve implantation (TAVI) have become available [6–8]. This procedure is currently considered to be the procedure of choice in the treatment of patients with AS and high surgical risk, especially in elderly individuals [6]. However, clinical trials evaluating the therapeutic role of TAVI in patients at intermediate surgical risk have shown non-inferiority between TAVI and SAVR in the primary endpoint [8, 9].

Type 2 Diabetes mellitus is strongly related to the in-hospital and short-term prognosis in patients with cardiovascular diseases needing surgical or invasive interventions [10, 11]. How T2DM might influence the treatment of AS has not been completely elucidated for SAVR or TAVI [9, 12–14]. In addition, the conflicting results of published clinical trials and multicenter studies led to the current research.

The aims of this study were: (1) to describe the use of aortic valve replacement procedures (TAVI and SAVR) among hospitalized patients with and without T2DM; and (2) to identify factors associated with in hospital mortality (IHM) among patients undergoing these procedures.

## Methods

We analyzed data from the Spanish National Hospital Discharge Database (SNHDD), between January 1, 2014 and December 31, 2015 for patients aged 40 years and over. This database covers more than 98% of Spanish hospital admissions and includes patient’s sex and date of birth, admission and discharge dates, diagnoses at discharge (up to 14), and procedures (up to 20) performed during the hospital stay [15].

We selected patients whose medical procedures included TAVI [International Classification of Diseases, Ninth Revision, Clinical Modification (ICD-9-CM) codes 35.05, 35.06] and SAVR (ICD-9-CM codes 35.21, 35.22). TAVI codes were included in the SNHDD in year 2014. If a patient had both procedures was excluded. Also those with other cardiac surgery procedures were excluded.

We stratified each cohort by diabetes status: T2DM (ICD-9-CM codes 250.x0, 250.x2) and no diabetes.

Clinical characteristics included information on overall comorbidities at the time of diagnosis, assessed by calculating the Charlson comorbidity index (CCI) [16]. The CCI was calculated excluding DM as a disease. We retrieved data about specific comorbidities, risk factors, procedures, and specific in-hospital postoperative complications. The conditions studied and the codes used to identify them according to the ICD-9-CM are shown in Additional file 1: Table S1.

We created a new variable “Any post procedure complication” that includes those patients who suffered infection and/or mediastinitis and/or functional disturbances following SAVR or TAVI.

Hospital outcome variables included in-hospital mortality (IHM) length of hospital stay (LOHS) and costs. IHM was defined as the proportion of patients who died during the admission.

### Statistical analysis

A descriptive statistical analysis was performed. Variables are expressed as proportions or as means with standards deviations (SDs). We compared specific comorbidities, risk factors, procedures, specific in-hospital postoperative complications, and in-hospital outcomes between T2DM patients and noT2DM patients in the TAVI cohort and in SAVR cohort. To compare continuous variables, we used the Student’s T test and Chi square test for proportions.

We performed two unconditional logistic regression analyses to identify the variables associated to in-hospital mortality as a binary outcome among all patients who underwent TAVI and SAVR. The variables included in the models were those with significant results in the bivariate analysis and those considered relevant in other investigations (obesity).

We also conducted two logistic regression models using “Any post procedure complication” as dependent variables to assess the effect of DM after controlling for other variables.

Estimates were expressed as odds ratios (OR) with their 95% confidence intervals.

All statistical analyses were performed using Stata version 10.1 (Stata, College Station, Texas, USA). Statistical significance was set at p < 0.05 (2-tailed).

### Ethical aspects

Data confidentiality was maintained at all times in accordance with Spanish legislation. Given the anonymous and mandatory nature of the dataset, it was not deemed necessary to obtain informed consent nor an ethics committee approval.

## Results

In 2014/15, we identified a total of 2141 and 16,013 patients who underwent TAVI (n = 715; 33.39% with T2DM) and SAVR (n = 4057; 25.33% with T2DM).

Table 1 shows the characteristics of hospital admission for patients who underwent the TAVI procedure in Spain, 2014/15. In our study, we found that 46.71% were women with diabetes compared with 52.66% without diabetes (p = 0.009). Patients with T2DM were significantly younger than those without diabetes (79.52 years vs. 81.36 years, respectively).

**Table 1:** Characteristics, comorbidities, risk factors, procedures, postoperative complications and in-hospital outcomes in patients underwent TAVI in Spain in 2014/15, according to type 2 diabetes status

	T2DM n = 715	No T2DM n = 1426	p value
Female sex, n (%)	334 (46.71)	751 (52.66)	0.009
Age, mean (SD)	79.52 (6.27)	81.36 (6.71)	< 0.01
Age groups, n (%)			
40–79 years	298 (41.68)	401 (28.12)	< 0.01
80–84 years	281 (39.3)	538 (37.73)	
≥ 85 years	136 (19.02)	487 (34.15)	
Charlson comorbidity index, mean (SD)^a^	1.22 (1)	1.03 (0.96)	< 0.01
Congestive heart failure, n (%)	204 (28.53)	374 (26.23)	0.257
Cerebrovascular disease, n (%)	46 (6.43)	73 (5.12)	0.211
Dementia, n (%)	5 (0.7)	6 (0.42)	0.395
Chronic pulmonary disease, n (%)	208 (29.09)	383 (26.86)	0.276
Renal disease, n (%)	195 (27.27)	269 (18.86)	< 0.01
Ischemic heart disease, n (%)	314 (43.92)	478 (33.52)	< 0.01
Atrial fibrillation, n (%)	221 (30.91)	508 (35.62)	0.030
Intermittent claudication, n (%)	12 (1.68)	28 (1.96)	0.646
Endocarditis, n (%)	1 (0.14)	1 (0.07)	0.618
Hypertension, n (%)	432 (60.42)	722 (50.63)	< 0.01
Lipid metabolism disorders, n (%)	363 (50.77)	509 (35.69)	< 0.01
Smoking, n (%)	130 (18.18)	213 (14.94)	0.054
Obesity, n (%)	123 (17.2)	111 (7.78)	< 0.01
Catheterization, n (%)	112 (15.66)	228 (15.99)	0.846
Computerized tomography of the torax, n (%)	51 (7.13)	104 (7.29)	0.893
Magnetic resonance imaging, n (%)	1 (0.14)	4 (0.28)	0.525
Pacemaker device implantation, n (%)	100 (13.99)	214 (15.01)	0.529
Cardioversion, n (%)	14 (1.96)	35 (2.45)	0.469
Balloon counterpulsation, n (%)	3 (0.42)	10 (0.7)	0.429
Percutaneous coronary interventions	30 (4.20)	66 (4.63)	0.648
Hemodialysis, n (%)	20 (2.8)	30 (2.1)	0.316
Red blood cell transfusion, n (%)	120 (16.78)	215 (15.08)	0.305
Infection, n (%)	13 (1.82)	35 (2.45)	0.348
Mediastinitis, n (%)	0 (0)	1 (0.07)	0.479
Functional disturbances following TAVI, n (%)	6 (0.84)	15 (1.05)	0.638
Any post procedure complication^b^	19 (2.66)	49 (3.44)	0.332
Mortality, n (%)	26 (3.64)	73 (5.12)	0.123
Length of stay, mean (SD)	12.54 (11.43)	12.83 (12.07)	0.603
Cost, mean (SD)	20,356 (8897)	20,983 (9623)	0.145

^a^CCI was calculated excluding DM

^b^Any post procedure complication included: infection; mediastinitis; and functional disturbances following TAVI

Diabetic patients who underwent a TAVI procedure had higher CCI mean values compared to those without T2DM (1.22 vs. 1.03, respectively; p < 0.01). Among patients with T2DM, the most common comorbidities according to the CCI included chronic pulmonary diseases (29.09%), congestive heart failure (28.53%), and renal disease (27.27%). Renal disease was significantly more frequent in patients with diabetes than in those without diabetes, as can been seen in Table 1.

Patients with T2DM had a higher incidence of ischemic heart disease (43.92% vs. 33.52%, p < 0.01), hypertension (60.42% vs. 50.63%, p < 0.01), lipid metabolism disorders (50.77% vs. 35.69%, p < 0.01), and obesity (17.2% vs. 7.78%, p < 0.01) than those without diabetes (Table 1). Atrial fibrillation was less prevalent among T2DM (30.91% vs. 35.62%; p = 0.030).

In patients who underwent TAVI, red blood cell transfusion, catheterization, and pacemaker device implantation were the most frequent procedures. We found no differences in IHM (3.64% in T2DM patients vs. 5.12% in non-T2DM patients, p = 0.603).

The characteristics, comorbidities, risk factors, procedures, postoperative complications, and in-hospital outcomes in patients with and without diabetes who underwent SAVR are shown in Table 2.

**Table 2:** Characteristics, comorbidities, risk factors, procedures, postoperative complications and in-hospital outcomes in patients underwent SAVR in Spain in 2014/15, according to type 2 diabetes status

	T2DM n = 4057	No T2DM n = 11,956	p value
Female sex, n (%)	1630 (40.18)	5061 (42.33)	0.016
Age, mean (SD)	72.47 (7.76)	70.36 (10.03)	< 0.01
Age groups, n (%)			
40–66 years	828 (20.41)	3562 (29.79)	< 0.01
67–75 years	1591 (39.22)	3961 (33.13)	
≥ 76 years	1638 (40.37)	4433 (37.08)	
Charlson comorbidity index, mean (SD)^a^	0.83 (0.9)	0.81 (0.88)	< 0.01
Congestive heart failure, n (%)	711 (17.53)	2229 (18.64)	0.112
Cerebrovascular disease, n (%)	284 (7)	682 (5.7)	0.003
Dementia, n (%)	13 (0.32)	17 (0.14)	0.023
Chronic pulmonary disease, n (%)	798 (19.67)	2295 (19.2)	0.508
Renal disease, n (%)	571 (14.07)	1085 (9.07)	< 0.01
Ischemic heart disease, n (%)	1626 (40.08)	3470 (29.02)	< 0.01
Atrial fibrillation, n (%)	1523 (37.54)	4778 (39.96)	0.006
Intermittent claudication, n (%)	67 (1.65)	80 (0.67)	< 0.01
Endocarditis, n (%)	128 (3.16)	549 (4.59)	< 0.01
Hypertension, n (%)	2731 (67.32)	6094 (50.97)	< 0.01
Lipid metabolism disorders, n (%)	1856 (45.75)	3447 (28.83)	< 0.01
Smoking, n (%)	1041 (25.66)	2920 (24.42)	0.115
Obesity, n (%)	729 (17.97)	1200 (10.04)	< 0.01
Catheterization, n (%)	523 (12.89)	1302 (10.89)	0.001
Computerized tomography of the torax, n (%)	149 (3.67)	629 (5.26)	< 0.01
Magnetic resonance imaging, n (%)	13 (0.32)	45 (0.38)	0.608
Pacemaker device implantation, n (%)	194 (4.78)	551 (4.61)	0.651
Cardioversion, n (%)	205 (5.05)	749 (6.26)	0.005
Balloon counterpulsation, n (%)	55 (1.36)	275 (2.3)	< 0.01
Percutaneous coronary interventions	41 (1.01)	70 (0.59)	0.005
Hemodialysis, n (%)	127 (3.13)	435 (3.64)	0.129
Red blood cell transfusion, n (%)	941 (23.19)	2806 (23.47)	0.721
Infection, n (%)	148 (3.65)	554 (4.63)	0.008
Mediastinitis, n (%)	10 (0.25)	46 (0.38)	0.197
Functional disturbances following SAVR, n (%)	22 (0.54)	101 (0.84)	0.057
Any post procedure complication^b^	170 (4.19)	659 (5.51)	0.001
Mortality, n (%)	177 (4.36)	754 (6.31)	< 0.01
Length of stay, mean (SD)	16.37 (13.77)	17.54 (17.27)	< 0.01
Cost, mean (SD)	23,667 (11,994)	24,659 (15,210)	< 0.001

^a^CCI was calculated excluding DM

^b^Any post procedure complication included: infection; mediastinitis; and functional disturbances following SAVR

Patients withT2DM were significantly older (72.47 ± 7.76 years vs. 70.36 ± 10.03 years, p < 0.01) and had higher frequencies of comorbidities according to the CCI (0.83 ± 0.9 vs. 0.81 ± 0.88, p < 0.01) than non-diabetic patients. Renal disease was significantly more common in patients with diabetes (14.07%) than in those without diabetes (9.07%).

Hypertension (67.32% vs. 50.97%, p < 0.01), lipid metabolism disorders (45.75% vs. 28.83%, p < 0.01), ischemic heart disease (40.08% vs. 29.02%, p < 0.01), obesity (17.97% vs. 10.04%, p < 0.01), and intermittent claudication (1.65% vs. 0.67%, p < 0.01) were significantly more prevalent in diabetic patients than in those without T2DM. However diabetic patients had a lower frequency of atrial fibrillation (37.54% vs. 39.96%, p = 0.006) and endocarditis (3.16% vs. 4.59%, p < 0.01) than non-T2DM patients (Table 2).

Catheterization rate and percutaneous coronary interventions were significantly higher in diabetic patients than in those without diabetes (12.89% vs. 10.89% and 1.01% vs. 0.59% respectively). However, computerized tomography of the thorax and balloon counterpulsation were significantly more frequent in non-T2DM patients than in diabetic patients, as can been seen in Table 2. Also postoperative infection (3.65% vs. 4.63%, p = 0.008) was lower in diabetic patients than in the non-T2DM group. The prevalence of “Any post procedure complication” was significantly higher among those without than with T2DM (5.51% vs. 4.19%; p = 0.001).

In the cohort of SAVR, mean LOHS was significantly lower in patients with T2DM than in non-diabetic patients (13.77 days vs. 17.27 days). As expected this was associated to a lower cost among T2DM patients (23,667 vs. 24,659 Euros; p < 0.001).

In hospital mortality was significantly lower in patients with T2DM (4.36% vs. 6.31%, p < 0.01) than in non-diabetic patients.

Table 3 shows the result of logistic regression analyses to assess the factors associated with IHM in all patients during hospital admission for TAVI and SAVR procedures. Comorbidities, computerized tomography of the thorax, cardioversion, hemodialysis, and red blood cell transfusion increased the risk of IHM in patients who underwent TAVI and SAVR.

**Table 3:** Logistic regression analysis of the factors associated with in-hospital mortality in all patients who underwent TAVI and SAVR in Spain, 2014/15

	OR (95% CI)
	TAVI (n = 2141)
Charlson comorbidity index^a^	1.60 (1.29–1.96)
Diabetes	0.60 (0.37–0.99)
Computerized tomography of the torax	1.85 (1.02–3.54)
Cardioversion	5.30 (2.42–11.60)
Hemodialysis	15.74 (8.08–30.64)
Red blood cell transfusion	1.96 (1.21–3.17)
Obesity	0.95 (0.55–2.26)

	SAVR (n = 16,013)
Female sex	1.33 (1.14–1.56)
Age groups (years)	
40–66	1
67–75	1.57 (1.26–1.96)
≥ 76	2.68 (2.17–3.31)
Charlson comorbidity index^a^	1.61 (1.49–1.74)
Atrial fibrillation	0.67 (0.57–0.79)
Diabetes	0.80 (0.66–0.96)
Endocarditis	2.13 (1.62–2.79)
Hypertension	0.64 (0.54–0.75)
Lipid metabolism disorders	0.81 (0.68–0.97)
Smoking	0.60 (0.48–0.76)
Computerized tomography of the torax	2.03 (1.57–2.62)
Pacemaker device implantation	0.68 (0.47–0.99)
Cardioversion	1.53 (1.17–2.00)
Balloon counterpulsation	11.11 (8.50–14.52)
Hemodialysis	9.98 (8.02–12.40)
Red blood cell transfusion	1.71 (1.46–1.99)
Postoperative infection	2.28 (1.75–2.97)
Length of stay	0.98 (0.97–0.99)
Obesity	0.92 (0.71–1.20)

^a^Charlson comorbidity index was calculated excluding DM

Among patients who underwent SAVR, endocarditis, balloon counterpulsation, and postoperative infection were associated with higher IHM. However, patients with atrial fibrillation, hypertension, lipid metabolism disorders, smoking, pacemaker device implantation, and higher LOHS had lower risk of IHM.

For both procedures, patients with T2DM had significantly lower mortality than patients without diabetes (adjusted OR 0.60; IC 95% 0.37–0.99 for TAVI and adjusted OR 0.80; IC 95% 0.66-0-96 for SAVR).

The results of the multivariable logistic regression showed no significant association of T2DM with suffering “Any post procedure complication” with adjusted OR’s of 0.80 (95% CI 0.45–1.44) for TAVI and 0.87 (95% CI 0.72–1.04) for SAVR (see Additional file 1: Table S2).

## Discussion

This study showed that T2DM does not increase in-hospital mortality in patients with AS requiring valvular replacement either through open surgery or transcatheter aortic valve implantation. Outcomes with TAVI in T2DM are conflicting in published reports [17, 18]. In a recent subanalysis, of the clinical trial PARTNER (Placement of Aortic Transcatheter Valves), mortality at 1 year follow up was higher in non-T2DM individuals [19]. However, a German registry showed that T2DM patients receiving TAVI had a worse prognosis with increase mortality in the short and long terms [20].

In Israel 443 patients (35.6% suffering DM) with severe AS undergoing TAVR were followed for 2 years and the results of the Kaplan–Meier survival analysis showed that DM was not associated with increased mortality [21].

Sun et al. [22] have published a recent meta-analysis showing that T2DM patients did not have an increase in mortality 30 days and 1 year after TAVI. A sub-analysis of the clinical trial PARTNER also showed a decrease of annual mortality in T2DM patients undergoing TAVI versus SAVR [19]. According to researchers, this tendency could be the result of the presence of obesity in the group of diabetic patients. Many studies have demonstrated obesity to have a protector effect on in-hospital mortality [23]. In our study, T2DM patients undergoing TAVI were more often obese compared to non diabetic patients. However, as can be seen in Table 3, after multivariable adjustment obesity was not associated to IHM in patients undergoing TAVI or SAVR in our investigation.

In our database, T2DM patients undergoing TAVI implantation were younger than the non-diabetic cases. The proportion of patients aged 85 years and more was significantly lower in the group of T2DM patients undergoing TAVI. This fact might have influenced on the prognostic results of diabetes in the study considering elderly patients with diabetes mellitus and established cardiovascular disease have higher mortality [24].

In cases undergoing SAVR, T2DM patients were found to have lower mortality than non-diabetic patients. These results differ from those reported by other authors who have found an increase in mortality in T2DM patients who underwent SAVR [14]. Despite an overall lower mortality in patients with DM, those diabetics undergoing SAVR had a higher mortality than diabetics undergoing TAVI. Ando et al. [14], in a recent study, demonstrated that diabetic patients with aortic stenosis undergoing TAVI (n = 5719) had lower in–hospital mortality compared to those undergoing open surgical replacement (N = 65,096). The lower in-hospital mortality in T2DM patients undergoing SAVR compared to non-diabetics might be multifactorial. Obesity was also more prevalent in T2DM patients undergoing SAVR and this might have contributed to the decrease of in-hospital mortality as previously mentioned [23].

Post-surgery infections and endocarditis were less frequent in T2DM patients undergoing open SAVR. It is well known that endocarditis worsens the prognosis after valvular intervention [25]. In our series, the multivariate analysis of SAVR cases showed that endocarditis and infections were factors independently associated with mortality. Of note, balloon counterpulsation was more often used in patients without T2DM and undergoing open surgery. This procedure is a marker of severity in post-surgical care and it is associated with an increase of mortality as observed in our study [26].

In our study we found that some results of the regression analysis for SAVR do not appear to clinically make sense including that atrial fibrillation, hypertension and smoking, lipid disorder were associated with lower IHM. In our opinion the possible explanation for this is a coding bias. Previous studies have found that people who codify may not record risk factors or not very severe conditions, such as atrial fibrillation, when other severe conditions are present [27, 28]. As a consequence of this patients with less severe conditions have risk factor and other diseases such as atrial fibrillation over codified. This over codification would result in a protective effect of these conditions.

The overall IHM in patients undergoing TAVI in our study was 4.62%. This figure is much higher than that reported by the FORWARD Study (1.9% at 30 days) [29]. The use of next-generation, self-expanding Evolut R THV and different selection criteria in the FORWARD Study may explain the differences [29].

The interpretation of our results, regarding the prognostic impact on survival of diabetes in patients undergoing TAVI or SAVR, may have some limitations. The degree of frailty and functional capacity of patients undergoing valve implantation was unknown. However, it is well known that fragile patients are at higher risk of complications after TAVI [30–32]. Gylcated hemoglobin, disease course and the antidiabetic treatment received are also factors influencing the prognosis after TAVI in T2DM patients. In this respect, Conrotto et al. [33] showed that diabetic patients under insulin regime and with poor glycemic control had higher mortality after TAVI compared to patients receiving oral antidiabetic treatment and better glycemic control. However, our study is based on an administrative database, so it lacks some relevant clinical parameters such as glycemic control, glycated hemoglobin, treatments during hospitalization or left ventricular ejection fraction. Absence of these parameters may affect the analysis and limit the generalizability of this study.

We did not compare TAVI versus SAVR as the clinical characteristics of patients in both groups was quite different. Individuals undergoing TAVI were in general older and with more comorbities compared to those undergoing SAVR. This could be explained from the fact that TAVI is mainly indicated in patients at high surgical risk in Spain. In a recent report from the Spanish National Society of Cardiology (Spain: coronary and structural heart interventions from 2010 to 2015) was stated that the 73.2% of patients undergoing TAVI were not elected for SAVR and were at very high surgical risk [34]. Information about surgical risk was not available in our database but we could infer that most patients undergoing TAVI were at high surgical risk. In future studies, it will be of interest to know the prognostic role of diabetes in patients at low or intermediate risk undergoing TAVI or SAVR.

It must be highlighted that infra-codification of TAVI could be possible in our database because this code was first introduced in year 2014-despite this, it seems that the frequency of TAVI implantation is lower than in other European countries [34]. Besides, in diabetic patients undergoing TAVI, the type of prosthesis and implantation pathway were not analyzed even though we know that transfemoral via is the most widely used (85% of cases in Spain in 2015) [34].

Unfortunately the SNHDD doesn’t include dates for the coded diagnosis. Therefore, it is not possible to know if diseases such as myocardial infarction, stroke, vascular complications, acute kidney injury among others were comorbid conditions present when the patients was admitted to the hospital or if appeared as a perioperative complication. Unknown differences in the incidence of these major complications could explain the lower IHM in T2DM patients compared to non-diabetics.

## Conclusions

Type 2 diabetes mellitus patients with AS undergoing a valvular replacement procedure through SAVR or TAVI did not have a worse prognosis compared to non-diabetic patients during hospitalization, showing lower IHM after multivariable adjustment. However, given the methodological limitations of administrative data more prospective studies and clinical trials aimed at evaluating the influence of these procedures in diabetic patients with AS are needed.

## Additional file

**Additional file 1: Table S1.** Comorbidities, risk factors, procedures and in-hospital postoperative complications with corresponding ICD-9-CM codes. **Table S2.** Logistic regression analysis of the factors associated with *“Any post procedure complication”* in all patients who underwent TAVI and SAVR in Spain, 2014/15.

## Abbreviations

AS: aortic stenosis
CCI: Charlson comorbidity index
ICD-9-CM: International Classification of Diseases, Ninth Revision, Clinical Modification
DM: diabetes mellitus
IHM: in hospital mortality
LOHS: length of hospital stay
SAVR: surgical aortic valve replacement
SNHDD: Spanish National Hospital Discharge Database
T2DM: type 2 diabetes mellitus
TAVI: transcatheter aortic valve implantation
