# Supplementary material for: Transcatheter aortic valve implantation and surgical aortic valve replacement among hospitalized patients with and without type 2 diabetes mellitus in Spain (2014–2015)
Source: Cardiovasc Diabetol. 2017 Nov 9;16:144. doi: 10.1186/s12933-017-0631-6 (PMC5679322; doi:10.1186/s12933-017-0631-6)
Supplement: Supplementary file 1 — Additional file 1: Table S1. Comorbidities, risk factors, procedures and in-hospital postoperative complications with corresponding ICD-9-CM codes. Table S2. Logistic regression analysis of the factors associated with “Any post procedure complication” in all patients who underwent TAVI and SAVR in Spain, 2014/15. [file 12933_2017_631_MOESM1_ESM.docx]

Table S1. Comorbidities, risk factors, procedures and in-hospital postoperative complications with corresponding ICD-9-CM codes.

|  | **ICD-9-CM codes** |
| --- | --- |
| Congestive heart failure | 398.91, 402.01, 402.11, 402.91,404.01, 404.03, 404.11,404.13, 404.91, 404.93,425.4–425.9, 428.x |
| Cerebrovascular disease | 362.34, 430.x–438.x |
| Dementia | 290.x, 294.1, 331.2 |
| Chronic pulmonary disease | 416.8, 416.9, 490.x–505.x,506.4, 508.1, 508.8 |
| Renal disease | 403.01, 403.11, 403.91, 404.02, 404.03, 404.12, 404.13, 404.92, 404.93, 582.x, 583.0–583.7, 585.x, 586.x, 588.0, V42.0, V45.1, V56.x |
| Ischemic heart disease | 410.x-414.x |
| Atrial fibrillation | 427.31 |
| Intermittent claudication | 443.9 |
| Endocarditis | 424.90, 421.9, 424.1, 421.0 |
| Hypertension | 401, 401.0, 401.1, 401.9 |
| Lipid metabolism disorders | 272.4 |
| Current smoking | 305.1, V15.82 |
| Obesity | 278.xx |
| Catheterization | 88.52-88.57 |
| Computerized tomography of the torax | 87.41 |
| Magnetic resonance imaging | 88.92 |
| Pacemaker device implantation | 37.8, 37.80-89 |
| Cardioversion | 99.62 |
| Balloon counterpulsation | 37.61 |
| Percutaneous coronary interventions | 36.06, 36.07, 36.09, 0.66. |
| Hemodialysis | 39.95, 54.98 |
| Red blood cell transfusion | 99.0, 99.00-09 |
| Postoperative infection | 998.5, 998.51, 998.59, 682.2, 682.9, 041.1, 041.10-12, 041.19, 85.0, 85.91, 83.44, 83.45, 83.49, 86.01, 86.04, 86.09, 86.22, 86.28 |
| Mediastinitis | 519.2 |
| Functional disturbances following SAVR or TAVI | 429.4 |

Table S2. Logistic regression analysis of the factors associated with *“Any post procedure complication”* in all patients who underwent TAVI and SAVR in Spain, 2014/15.

|  | | Odds Ratio (95% CI) |
| --- | --- | --- |
|  | | **TAVI (n=2141)** |
| Age groups | 40-79 years |  |
|  | 80-84 years | 1.39 (0.74-2.61) |
|  | ≥85 years | 1.10(0.52-2.31) |
| Charlson comorbidity index* | | 0.89(0.67-1.19) |
| Sex | Female | 1.21(0.71-2.08) |
| T2MD | | 0.80(0.45-1.44) |
|  | | **SAVR (n=16013)** |
|  | | Odds Ratio (95% CI) |
| Age groups | 40-66 years | 1 |
|  | 67-75 years | 1.02(0.88-1.19) |
|  | ≥76 | 1.09(0.96-1.26) |
| CCI* | | 1.11(1.03-1.21) |
| Sex | Female | 0.90(0.77-1.06) |
| T2DM | | 0.87(0.72-1.04) |

* Charlson comorbidity index was calculated excluding DM.
